# Barriers to Timely Seeking of Breast Cancer Care Among Palestinian Women: A Cross-Sectional Study

**DOI:** 10.1200/GO.23.00373

**Published:** 2024-02-22

**Authors:** Mohamedraed Elshami, Malak Ayman Qawasmi, Roba Jamal Ghithan, Ibrahim Al-Slaibi, Mohammed Alser, Nouran Ramzi Shurrab, Islam Osama Ismail, Ibtisam Ismail Mahfouz, Aseel AbdulQader Fannon, Mona Radi Hawa, Narmeen Giacaman, Manar Ahmaro, Heba Mahmoud Okshiya, Rula Khader Zaatreh, Wafa Aqel AbuKhalil, Faten Darwish Usrof, Noor Khairi Melhim, Ruba Jamal Madbouh, Hala Jamal Abu Hziema, Raghad Abed-Allateef Lahlooh, Sara Nawaf Ubaiat, Nour Ali Jaffal, Reem Khaled Alawna, Salsabeel Naeem Abed, Bessan Nimer Abuzahra, Aya Jawad Abu Kwaik, Mays Hafez Dodin, Raghad Othman Taha, Dina Mohammed Alashqar, Roaa Abd-alfattah Mobarak, Tasneem Smerat, Shurouq I. Albarqi, Nasser Abu-El-Noor, Bettina Bottcher

**Affiliations:** ^1^ Division of Surgical Oncology, Department of Surgery, University Hospitals Cleveland Medical Center, Cleveland, OH; ^2^ Ministry of Health, Gaza, Palestine; ^3^ Department of Medical Laboratory Sciences, Hebron University, Hebron, Palestine; ^4^ Faculty of Medicine, Al-Quds University, Jerusalem, Palestine; ^5^ Almakassed Hospital, Jerusalem, Palestine; ^6^ The United Nations Relief and Works Agency for Palestine Refugees in the Near East, Gaza, Palestine; ^7^ Faculty of Medicine, Al Azhar University-Gaza, Gaza, Palestine; ^8^ Faculty of Medicine, Islamic University of Gaza, Gaza, Palestine; ^9^ Tulkarem Governmental Hospital, Tulkarem, Palestine; ^10^ Al-Shifa Hospital, Gaza, Palestine; ^11^ Caritas Baby Hospital, Bethlehem, Palestine; ^12^ Faculty of Health Sciences Master of Medical Laboratory Sciences, Islamic University of Gaza, Gaza, Palestine; ^13^ Department of Pharmacy, An-Najah National University, Nablus, Palestine; ^14^ Faculty of Dentistry, Al-Quds University, Jerusalem, Palestine; ^15^ Faculty of Medicine and Health Sciences, Palestine Polytechnic University, Hebron, Palestine; ^16^ Faculty of Pharmacy, Al-Azhar University of Gaza, Gaza, Palestine; ^17^ Faculty of Nursing, Islamic University of Gaza, Gaza, Palestine

## Abstract

**PURPOSE:**

Examining the association of breast cancer (BC) symptom awareness with time to help seeking and exploring barriers to timely presentation may enhance the effectiveness of BC awareness campaigns and early detection efforts. This study aimed to assess the anticipated time for seeking medical advice when experiencing a potential BC symptom among women in Palestine and to identify their barriers to early presentation.

**MATERIALS AND METHODS:**

A convenience sampling method was used to recruit adult women from hospitals, primary health care facilities, and public areas across 11 governorates in Palestine. A translated-into-Arabic version of the validated BC Awareness Measure was used. The questionnaire consisted of three sections: sociodemographic information, recognition of 13 BC symptoms and reporting time for seeking medical advice, and barriers to early presentation.

**RESULTS:**

A total of 5,257 questionnaires were included. The proportion of participants who would seek medical advice immediately varied on the basis of the nature of BC symptoms. For symptoms related to the breast, the proportion ranged from 25.7% for redness of the breast skin to 53.5% for a lump or thickening in the breast. For symptoms related to the nipple, the proportion ranged from 30.7% for nipple rash to 48.0% for discharge or bleeding from the nipple. Exhibiting good BC symptom awareness was associated with a higher likelihood of seeking medical advice within a week for all BC symptoms. Emotional barriers were the most frequently reported barriers. There was no association between increasing levels of BC awareness and reporting fewer or more barriers.

**CONCLUSION:**

The nature of BC symptoms had an impact on help-seeking behaviors. Participants with good BC symptom awareness were more likely to seek medical advice earlier.

## INTRODUCTION

Breast cancer (BC) was the most frequently diagnosed cancer with an incidence of 2.8 million cases and an estimated number of deaths of about 685,000 in 2020 worldwide.^1^ In the Eastern Mediterranean region, BC is the leading cause of cancer-related deaths among women in most countries.^2^ Notably, the incidence of BC in the region is expected to duplicate by 2030.^3^ In Palestine, BC is the most prevalent cancer in women, with an incidence rate of 36.2 cases per 100,000 women.^4^ BC ranks third in cancer-related mortality in Palestine, following lung and colorectal cancers, and accounts for 11.3% of total deaths.^5^

**CONTEXT**

**Key Objective**
This study assessed the anticipated time for seeking medical advice when experiencing a potential symptom of breast cancer (BC) and examined its association with BC symptom awareness. It also identified Palestinian women's barriers to early presentation.
**Knowledge Generated**
The proportion of participants who would seek medical advice immediately varied on the basis of the nature of BC symptoms. Good BC symptom awareness was associated with a higher likelihood of seeking medical advice within a week for all BC symptoms. Emotional barriers were the most frequently reported barriers to early presentation. There was no association between higher BC awareness and reporting fewer or more barriers.
**Relevance**
Our results demonstrate the importance of developing and implementing appropriate educational interventions that aim to promote BC awareness. Improving BC symptom awareness and mitigating barriers to early presentation might further facilitate timely diagnosis of BC and eventually decrease overall morbidity and mortality in Palestine.

Previous studies have shown what nature of BC symptoms was associated with the time interval to seek medical attention.^6,7^ Moodley et al found that patients experiencing nonlump symptoms had a longer delay, presenting between 1 week and 2 months later to health care professionals from noticing the symptoms. This delay was attributed to their perception that these symptoms were not indicative of a serious condition.^6^ Furthermore, Ströbele et al found that only 30% of study participants sought medical advice for breast-related symptoms. The major reasons for this small proportion were a lack of significant impact on daily life and inadequate awareness of BC symptoms, leading most participants to fail to recognize their own symptoms.^7^

Early detection of BC plays a vital role in reducing morbidity and mortality rates. Improving BC symptom awareness and promoting early help-seeking behaviors are essential in achieving this.^4,8,9^ However, various barriers can hinder women from seeking medical attention promptly, falling into categories such as emotional, service-related, and practical barriers.^10^ A study conducted in the Gaza Strip in 2018 found that emotional barriers were the most frequently reported obstacles among participants.^10^ Exploring these barriers in Palestine can contribute to enhancing the effectiveness of BC awareness campaigns and early detection efforts. By reducing delays in help seeking and facilitating earlier diagnosis, such initiatives may have the potential to positively affect treatment, outcomes, and prognosis.^4^

This national study aimed to examine (1) the anticipated time for seeking medical advice when experiencing a potential symptom of BC, (2) the association between BC symptom awareness and the anticipated time for seeking medical advice, and (3) Palestinian women's barriers to early presentation.

## MATERIALS AND METHODS

### Study Design and Population

This was a national cross-sectional study conducted from July 2019 to March 2020. Adult (age 18 years or older) Palestinian women were targeted. Data collection took place in the two main geographical areas of Palestine, which are the Gaza Strip (five governorates) and the West Bank and Jerusalem (WBJ; 11 governorates).^5^ Adult women residing in 11 governorates across Palestine (four governorates in the Gaza Strip and seven in the WBJ) were invited to participate. Excluded from participation in this study were women with non-Palestinian nationality, visitors to oncology departments, women who worked or studied in a health-related field, and those who were unable to complete the questionnaire.

### Sampling Methods

The recruitment of eligible women for this study was conducted through convenience sampling. Recruitment took place at medical facilities (ie, governmental hospitals and primary health care centers) and public spaces, with the aim of achieving a broad representation of Palestinian community.^10-21^ Participants were recruited from public spaces that included malls, markets, gardens, restaurants, churches, mosques, and public transport hubs.

### Validation and Piloting of Questionnaire

For data collection purposes, a modified version of the validated Breast Cancer Awareness Measure (BCAM) was used as the assessment tool.^8^ The original BCAM questionnaire in English was first translated into Arabic by two bilingual experts and subsequently translated back into English by two different bilingual experts. The Arabic version of BCAM was then evaluated by five experts specialized in BC, public health, and survey design to ensure content validity and accurate translation. To assess the clarity of the questions in the Arabic version of BCAM, a pilot study involving 35 participants was conducted. The responses from the pilot study were not included in the final analysis. To measure the internal consistency of the Arabic BCAM, Cronbach's alpha coefficient was computed, resulting in a satisfactory value of .75.

### Data Collection and Measurement Tool

The BCAM questionnaire comprised three sections. The first section included sociodemographic information, including age, age at menarche, highest level of education, employment status, monthly income, marital status, place of residency, presence of chronic diseases, knowing someone diagnosed with cancer, and site of data collection. The second section assessed BC symptom awareness and the estimated time women would take to seek medical consultation for each symptom. A total of 13 BC symptoms were evaluated, with 11 symptoms adapted from the original BCAM.^8^ Additionally, two new symptoms, namely extreme generalized fatigue and unexplained weight loss, were included on the basis of previous studies.^10,11,13^ To minimize arbitrary answers to questionnaire questions, a 5-point Likert scale (ranging from 1 = strongly disagree to 5 = strongly agree) was used instead of the original yes, no, and I do not know responses used in the original BCAM. The third section explored perceived barriers to early presentation, which were categorized into three categories: emotional, service-related, and practical barriers.

The electronic tool Kobo Toolbox (Cambridge, MA) was used for data collection.^22^ This secure tool can be used both online and offline and is compatible with smartphones. The participants were invited to take part in face-to-face interviews, during which they completed the questionnaire. Female data collectors, who had a background in the medical field, received specialized training on how to effectively use the Kobo Toolbox. They were also trained on approaching potential participants in waiting areas of hospitals, primary health care centers, and public spaces on a daily basis. The inclusion of female data collectors aimed to minimize potential embarrassment among women when answering sensitive questions. Ensuring privacy was a crucial aspect of the training provided, and the interviews were conducted in private settings at designated locations.

### Statistical Analysis

In Palestine, women are recommended to undergo BC screening starting at the age of 40 years.^10^ Consequently, the continuous variable of age was divided into two categories using this threshold: 18-39 years and  40 years or older. Similarly, the monthly income variable was categorized into two groups: <1,450 New Israeli Shekel (NIS) and ≥1,450 NIS. This categorization was based on the minimum wage in Palestine, which is 1,450 NIS, approximately equivalent to $450 (US dollars).^23^

The median and IQR were used to describe continuous variables that did not follow a normal distribution. Conversely, frequencies and percentages were used to describe categorical variables. To compare the baseline characteristics of participants recruited from the Gaza Strip and those recruited from the WBJ, the Kruskal-Wallis test was used for continuous variables while Pearson chi-square test was used for categorical variables.

BC symptoms were categorized into three categories: (1) breast symptoms, (2) nipple symptoms, and (3) other symptoms. Furthermore, the anticipated time to seek medical advice for a possible BC symptom was categorized into four categories: immediately (<24 hours), ≤7 days, >7 days, and never. The anticipated time was described using frequencies and percentages and was compared between participants from the Gaza Strip and those from the WBJ using Pearson chi-square test.

Consistent with previous reporting,^10-21^ one point was given for each correctly identified BC symptom, resulting in a total awareness score ranging from 0 to 13. The total awareness score was further categorized into three levels: poor awareness (0-4), fair awareness (5-9), and good awareness (10-13) on the basis of the number of symptoms recognized. Multivariable logistic regression was used to examine the association between BC symptom awareness level and anticipated time to seek medical advice for a possible BC symptom. The multivariable analysis adjusted for age group, menarche, educational level, employment status, monthly income, marital status, place of residency, presence of a chronic disease, knowing someone with cancer, and site of data collection. This model was determined a priori on the basis of previous studies.^10,13,24-29^

Consistent with the original BCAM,^8^ barriers to early presentation were classified into three categories: emotional, service-related, and practical barriers. The reported barriers were described using frequencies and percentages, and were compared between participants recruited from the Gaza Strip and those recruited from the WBJ using Pearson chi-square test. To dichotomize the number of displayed barriers (overall, emotional, service, and practical), the median number of barriers in each category was used as a cutoff. To investigate the association between BC symptom awareness level and the presence of at least the median number of barriers to early presentation, multivariable logistic regression analysis was used. The same aforementioned multivariable model was used.

Missing data were hypothesized to occur completely at random and were handled using a complete case analysis approach. Data were analyzed using Stata software version 17.0 (StataCorp, College Station, TX).

### Ethics Approval and Consent to Participate

This study received ethical approval from the Declaration of Helsinki Committee in the Gaza Strip, which is a committee under the Ministry of Health responsible for granting study approvals. Additionally, this study was approved by the Ethics Committee of the Islamic University of Gaza and the Human Resources Development department at the Palestinian Ministry of Health. Written informed consent was obtained from all study participants before conducting the interviews. Participants were provided with a comprehensive explanation of the study, emphasizing that their participation was voluntary and would not have any impact on the medical care they receive. All study methods were performed in accordance with relevant guidelines and regulations. Data confidentiality was maintained throughout the study.

## RESULTS

### Participant Characteristics

A total of 5,434 participants completed the questionnaire of 6,269 approached, resulting in a response rate of 86.7%. Among them, 5,257 questionnaires met the inclusion criteria and were included in the final analysis: 2,551 were from the Gaza Strip and 2,706 were from the WBJ. A total of 13 questionnaires did not meet the inclusion criteria, and 164 questionnaires had missing data. Participants from the WBJ were older, had higher monthly income, and suffered more frequently from chronic diseases than participants from the Gaza Strip (table 1). Only 2,191 participants (41.7%) demonstrated good awareness of BC symptoms. Participants from the Gaza Strip were more likely than participants from the WBJ to display good awareness of BC symptoms (46.9% *v* 36.7%).

**TABLE 1:** Characteristics of Study Participants

Characteristic	Total (N = 5,257)	Gaza Strip (n = 2,551)	WBJ (n = 2,706)	*P*
Awareness of BC symptoms, No. (%)				<.001
Poor	559 (10.6)	256 (10.0)	303 (11.2)	
Fair	2,507 (47.7)	1,098 (43.0)	1,409 (52.1)	
Good	2,191 (41.7)	1,197 (46.9)	994 (36.7)	
Age, years, median (IQR)	31.0 (24.0-43.0)	30.0 (24.0-40.0)	33.0 (24.0-45.0)	<.001
Age group, years, No. (%)				<.001
18-39	3,615 (68.8)	1,859 (72.9)	1,756 (64.9)	
40 years or older	1,642 (31.2)	692 (27.1)	950 (35.1)	
Menarche, No. (%)				.003
Normal (11-15 years)	4,608 (87.7)	2,237 (87.7)	2,371 (87.6)	
Early (≤10 years)	72 (1.4)	21 (0.8)	51 (1.9)	
Late (≥16 years)	577 (10.9)	293 (11.5)	284 (10.5)	
Educational level, No. (%)				.46
Secondary or below	3,030 (57.6)	1,457 (57.1)	1,573 (58.1)	
Postsecondary	2,227 (42.4)	1,094 (42.9)	1,133 (41.9)	
Occupation, No. (%)				<.001
Unemployed/housewife	3,568 (67.9)	1,868 (73.2)	1,700 (62.8)	
Employed	1,052 (20.0)	380 (14.9)	672 (24.9)	
Retired	13 (0.3)	5 (0.2)	8 (0.3)	
Student	624 (11.8)	298 (11.7)	326 (12.0)	
Monthly income ≥1,450 NIS, No. (%)	3,055 (58.1)	716 (28.1)	2,339 (86.4)	<.001
Having a chronic disease, No. (%)	1,058 (20.1)	397 (15.6)	661 (24.4)	<.001
Knowing someone with cancer, No. (%)	2,520 (47.9)	1,083 (42.5)	1,437 (53.1)	<.001
Marital status, No. (%)				<.001
Single	1,301 (24.7)	626 (24.5)	675 (24.9)	
Married	3,658 (69.6)	1,812 (71.0)	1,846 (68.2)	
Divorced/widowed	298 (5.7)	113 (4.4)	185 (6.8)	
Site of data collection, No. (%)				<.001
Public spaces	1,821 (34.6)	809 (31.7)	1,012 (37.4)	
Hospitals	2,116 (40.3)	919 (36.0)	1,197 (44.2)	
Primary health care centers	1,320 (25.1)	823 (32.3)	497 (18.4)	

Abbreviations: BC, breast cancer; NIS, New Israeli Shekel; WBJ, West Bank and Jerusalem.

### Immediate Seeking of Medical Advice for a Possible BC Symptom

About half of the participants (n = 2,810, 53.5%) reported that they would seek medical advice immediately for lump or thickening in the breast (table 2). The proportion was lower for other breast symptoms. About one half reported that they would seek medical advice immediately for discharge or bleeding from the nipple (n = 2,522, 48.0%) or lump or thickening under the armpit (n = 2,514, 47.8%). Less than one third reported immediate seeking of medical advice for all other BC symptoms. Women from the WBJ were more likely than women from the Gaza Strip to immediately visit a doctor if they recognized any possible BC symptom.

**TABLE 2:** Summary of the Reported Time to Seek Medical Advice for a Possible BC Symptom

Category of Symptoms	BC Sign/Symptom	Total (N = 5,257), n (%)	Gaza Strip (n = 2,551), n (%)	WBJ (n = 2,706), n (%)	*P*
Breast symptoms	A lump or thickening in the breast				<.001
	Immediately	2,810 (53.5)	1,207 (47.3)	1,603 (59.2)	
	≤7 days	2,205 (41.9)	1,233 (48.3)	972 (35.9)	
	>7 days	112 (2.1)	40 (1.6)	72 (2.7)	
	Never	130 (2.5)	71 (2.8)	59 (2.2)	
	Puckering or dimpling of the breast skin				<.001
	Immediately	1,658 (31.5)	738 (28.9)	920 (34.0)	
	≤7 days	2,313 (44.0)	1,318 (51.7)	995 (36.8)	
	>7 days	140 (2.7)	60 (2.4)	80 (3.0)	
	Never	1,146 (21.8)	435 (17.1)	711 (26.3)	
	Pain in one of the breasts or armpits				<.001
	Immediately	1,520 (28.9)	664 (26.0)	856 (31.6)	
	≤7 days	2,567 (48.8)	1,428 (56.0)	1,139 (42.1)	
	>7 days	243 (4.6)	86 (3.4)	157 (5.8)	
	Never	927 (17.6)	373 (14.6)	554 (20.5)	
	Redness of the breast skin				<.001
	Immediately	1,351 (25.7)	607 (23.8)	744 (27.5)	
	≤7 days	2,275 (43.3)	1,310 (51.4)	965 (35.7)	
	>7 days	159 (3.0)	71 (2.8)	88 (3.3)	
	Never	1,472 (28.0)	563 (22.1)	909 (33.6)	
Nipple symptoms	Discharge or bleeding from the nipple				<.001
	Immediately	2,522 (48.0)	1,109 (43.5)	1,413 (52.2)	
	≤7 days	2,390 (45.5)	1,289 (50.5)	1,101 (40.7)	
	>7 days	89 (1.7)	33 (1.3)	56 (2.1)	
	Never	256 (4.9)	120 (4.7)	136 (5.0)	
	Pulling in of the nipple				<.001
	Immediately	1,672 (31.8)	779 (30.5)	893 (33.0)	
	≤7 days	2,332 (44.4)	1,293 (50.7)	1,039 (38.4)	
	>7 days	167 (3.2)	65 (2.5)	102 (3.8)	
	Never	1,086 (20.7)	414 (16.2)	672 (24.8)	
	Change in the position of the nipple				<.001
	Immediately	1,669 (31.7)	763 (29.9)	906 (33.5)	
	≤7 days	2,436 (46.3)	1,327 (52.0)	1,109 (41.0)	
	>7 days	179 (3.4)	68 (2.7)	111 (4.1)	
	Never	973 (18.5)	393 (15.4)	580 (21.4)	
	Nipple rash				<.001
	Immediately	1,616 (30.7)	707 (27.7)	909 (33.6)	
	≤7 days	2,405 (45.7)	1,385 (54.3)	1,020 (37.7)	
	>7 days	121 (2.3)	49 (1.9)	72 (2.7)	
	Never	1,115 (21.2)	410 (16.1)	705 (26.1)	
Other symptoms	Lump or thickening under the armpit				<.001
	Immediately	2,514 (47.8)	1,058 (41.5)	1,456 (53.8)	
	≤7 days	2,278 (43.3)	1,256 (49.2)	1,022 (37.8)	
	>7 days	137 (2.6)	50 (2.0)	87 (3.2)	
	Never	328 (6.2)	187 (7.3)	141 (5.2)	
	Changes in the shape of the breast or nipple				<.001
	Immediately	1,873 (35.6)	801 (31.4)	1,072 (39.6)	
	≤7 days	2,377 (45.2)	1,348 (52.8)	1,029 (38.0)	
	>7 days	161 (3.1)	71 (2.8)	90 (3.3)	
	Never	846 (16.1)	331 (13.0)	515 (19.0)	
	Changes in the size of the breast or nipple				<.001
	Immediately	1,808 (34.4)	762 (29.9)	1,046 (38.7)	
	≤7 days	2,280 (43.4)	1,301 (51.0)	979 (36.2)	
	>7 days	169 (3.2)	70 (2.7)	99 (3.7)	
	Never	1,000 (19.0)	418 (16.4)	582 (21.5)	
	Unexplained weight loss				<.001
	Immediately	864 (16.4)	271 (10.6)	593 (21.9)	
	≤7 days	1,603 (30.5)	893 (35.0)	710 (26.2)	
	>7 days	594 (11.3)	267 (10.5)	327 (12.1)	
	Never	2,196 (41.8)	1,120 (43.9)	1,076 (39.8)	
	Extreme generalized fatigue				<.001
	Immediately	703 (13.4)	298 (11.7)	405 (15.0)	
	≤7 days	1,958 (37.2)	1,077 (42.2)	881 (32.6)	
	>7 days	241 (4.6)	108 (4.2)	133 (4.9)	
	Never	2,355 (44.8)	1,068 (41.9)	1,287 (47.6)	

Abbreviations: BC, breast cancer; WBJ, West Bank and Jerusalem.

### Association Between BC Symptom Awareness and No Seeking of Medical Advice

Among women who exhibited a fair BC symptom awareness, there was an associated decrease in the likelihood of not seeking medical advice for each BC symptom (table 3). Moreover, among women who displayed a good BC symptom awareness, there was a further associated decrease in the likelihood of not seeking medical advice for all BC symptoms.

**TABLE 3:** Multivariable Logistic Regression Analyzing the Association Between No Seeking of Medical Advice and Awareness of BC Symptoms

Awareness of BC Symptoms	(1) Breast Symptoms							
	A Lump or Thickening in the Breast		Pain in One of the Breasts or Armpits		Puckering or Dimpling of the Breast Skin		Redness of the Breast Skin	
	AOR (95% CI)	*P*	AOR (95% CI)	*P*	AOR (95% CI)	*P*	AOR (95% CI)	*P*
Poor	Ref	Ref	Ref	Ref	Ref	Ref	Ref	Ref
Fair	0.34 (0.22 to 0.51)	<.001	0.50 (0.40 to 0.62)	<.001	0.56 (0.46 to 0.68)	<.001	0.66 (0.54 to 0.80)	<.001
Good	0.15 (0.09 to 0.25)	<.001	0.36 (0.29 to 0.45)	<.001	0.25 (0.20 to 0.31)	<.001	0.30 (0.24 to 0.37)	<.001

Awareness of BC Symptoms	(2) Nipple Symptoms							
	Discharge or Bleeding From the Nipple		Nipple Rash		Change in the Position of the Nipple		Pulling in of the Nipple	
	AOR (95% CI)	*P*	AOR (95% CI)	*P*	AOR (95% CI)	*P*	AOR (95% CI)	*P*
Poor	Ref	Ref	Ref	Ref	Ref	Ref	Ref	Ref
Fair	0.39 (0.28 to 0.53)	<.001	0.67 (0.55 to 0.82)	<.001	0.67 (0.55 to 0.83)	<.001	0.77 (0.63 to 0.95)	.013
Good	0.19 (0.13 to 0.27)	<.001	0.24 (0.19 to 0.30)	<.001	0.33 (0.26 to 0.41)	<.001	0.32 (0.26 to 0.41)	<.001

Awareness of BC Symptoms	(3) Other Symptoms									
	Lump or Thickening Under the Armpit		Changes in the Shape of the Breast or Nipple		Changes in the Size of the Breast or Nipple		Unexplained Weight Loss		Extreme Generalized Fatigue	
	AOR (95% CI)	*P*	AOR (95% CI)	*P*	AOR (95% CI)	*P*	AOR (95% CI)	*P*	AOR (95% CI)	*P*
Poor	Ref	Ref	Ref	Ref	Ref	Ref	Ref	Ref	Ref	Ref
Fair	0.48 (0.36 to 0.64)	<.001	0.52 (0.42 to 0.64)	<.001	0.54 (0.44 to 0.66)	<.001	0.59 (0.49 to 0.72)	<.001	0.80 (0.66 to 0.97)	.021
Good	0.16 (0.11 to 0.23)	<.001	0.17 (0.14 to 0.22)	<.001	0.17 (0.13 to 0.21)	<.001	0.35 (0.29 to 0.43)	<.001	0.47 (0.39 to 0.57)	<.001

NOTE. All analyses were adjusted for age group, menarche, educational level, occupation, monthly income, marital status, residency, having a chronic disease, knowing someone with cancer, and site of data collection. The outcome in all models is binary, where answers with never were considered as yes and all other answers were considered as no.

Abbreviations: AOR, adjusted odds ratio; BC, breast cancer; Ref, reference.

### Association Between BC Symptom Awareness and Seeking Medical Advice Within a Week

The likelihood of seeking medical advice within a week for each of the BC symptoms was higher among women who displayed fair awareness of BC symptoms except for extreme generalized fatigue, compared with women with poor BC symptom awareness (table 4). In addition, there was a further associated increase in the likelihood of seeking medical advice within a week for all BC symptoms among women who displayed good awareness of BC symptoms.

**TABLE 4:** Multivariable Logistic Regression Analyzing the Association Between Seeking Medical Advice Within a Week and Awareness of BC Symptoms

Awareness of BC Symptoms	(1) Breast Symptoms							
	A Lump or Thickening in the Breast		Pain in One of the Breasts or Armpits		Puckering or Dimpling of the Breast Skin		Redness of the Breast Skin	
	AOR (95% CI)	*P*	AOR (95% CI)	*P*	AOR (95% CI)	*P*	AOR (95% CI)	*P*
Poor	Ref	Ref	Ref	Ref	Ref	Ref	Ref	Ref
Fair	2.04 (1.47 to 2.85)	<.001	1.76 (1.44 to 2.17)	<.001	1.77 (1.46 to 2.15)	<.001	1.49 (1.23 to 1.81)	<.001
Good	4.10 (2.78 to 6.07)	<.001	2.52 (2.03 to 3.14)	<.001	4.08 (3.30 to 5.06)	<.001	3.26 (2.66 to 4.01)	<.001

Awareness of BC Symptoms	(2) Nipple Symptoms							
	Discharge or Bleeding From the Nipple		Nipple Rash		Change in the Position of the Nipple		Pulling in of the Nipple	
	AOR (95% CI)	*P*	AOR (95% CI)	*P*	AOR (95% CI)	*P*	AOR (95% CI)	*P*
Poor	Ref	Ref	Ref	Ref	Ref	Ref	Ref	Ref
Fair	2.23 (1.68 to 2.96)	<.001	1.43 (1.17 to 1.74)	<.001	1.51 (1.23 to 1.85)	<.001	1.28 (1.05 to 1.57)	.015
Good	4.14 (2.98 to 5.75)	<.001	3.83 (3.07 to 4.78)	<.001	2.80 (2.25 to 3.49)	<.001	2.90 (2.33 to 3.60)	<.001

Awareness of BC Symptoms	(3) Other Symptoms									
	Lump or Thickening Under the Armpit		Changes in the Shape of the Breast or Nipple		Changes in the Size of the Breast or Nipple		Unexplained Weight Loss		Extreme Generalized Fatigue	
	AOR (95% CI)	*P*	AOR (95% CI)	*P*	AOR (95% CI)	*P*	AOR (95% CI)	*P*	AOR (95% CI)	*P*
Poor	Ref	Ref	Ref	Ref	Ref	Ref	Ref	Ref	Ref	Ref
Fair	1.87 (1.44 to 2.42)	<.001	1.81 (1.48 to 2.22)	<.001	1.71 (1.41 to 2.09)	<.001	1.56 (1.28 to 1.91)	<.001	1.20 (0.99 to 1.45)	.10
Good	4.49 (3.31 to 6.09)	<.001	5.09 (4.03 to 6.43)	<.001	5.04 (4.03 to 6.31)	<.001	2.39 (1.95 to 2.92)	<.001	2.03 (1.67 to 2.47)	<.001

NOTE. All analyses were adjusted for age group, menarche, educational level, occupation, monthly income, marital status, residency, having a chronic disease, knowing someone with cancer, and site of data collection. The outcome in all models is binary, where answers with 1-7 days were considered as yes and all other answers were considered as no.

Abbreviations: AOR, adjusted odds ratio; BC, breast cancer; Ref, reference.

### Barriers to Early Presentation and Lack of Association With BC Symptom Awareness

The study participants were asked if they would delay the visit to the doctor when they recognized a possible BC symptom and 508 (9.3%) answered with yes, where at least one barrier to early presentation was reported. Overall, the most common barriers were emotional with feeling worried about what a doctor might find (n = 281, 55.3%) and feeling scared (n = 278, 54.7%) being the most frequently reported barriers (Table 5). The most commonly reported service barrier was small number of female doctors in the Palestinian Ministry of Health facilities (n = 176, 34.6%). The most commonly reported practical barriers were participants would think that symptom is not something important to see the doctor for (n = 230, 45.3%) and too busy to go to the doctor (n = 225, 44.3%). All these findings were consistent in both the Gaza Strip and the WBJ.

**TABLE 5:** Summary of the Reported Barriers to Early Presentation Among Study Participants

Category	Barrier	Total (N = 508), No. (%)	Gaza Strip (n = 214), No. (%)	WBJ (n = 294), No. (%)	*P*
Emotional	Feeling worried about what a doctor might find	281 (55.3)	118 (55.1)	163 (55.4)	.99
	Feeling scared	278 (54.7)	116 (54.2)	162 (55.1)	.86
	Would not feel comfortable to be examined by a male doctor	255 (50.2)	103 (48.1)	152 (51.7)	.47
	Disliking the visit to health care facilities	240 (47.2)	101 (47.2)	139 (47.3)	.99
	Would prefer not to know the reason of the symptom	200 (39.4)	70 (32.7)	130 (44.2)	.010
	Embarrassment	195 (38.4)	78 (36.4)	117 (39.8)	.46
	God is the ultimate healer so there in no need to go to the doctor	194 (38.2)	89 (41.6)	105 (35.7)	.20
	Would not trust the doctor	122 (24.0)	55 (25.7)	67 (22.8)	.46
	Would not feel confident talking about symptom with	89 (17.5)	34 (15.9)	55 (18.7)	.48
Service	The small number of female doctors in the Palestinian Ministry of Health facilities	176 (34.6)	87 (40.7)	89 (30.3)	.018
	Difficulty making an appointment with the doctor	127 (25.0)	57 (26.6)	70 (23.8)	.47
	Feeling worried about wasting doctor's time	98 (19.3)	45 (21.0)	53 (18.0)	.43
	Difficulty talking to doctor	66 (13.0)	23 (10.7)	43 (14.6)	.23
Practical	Would think that symptom is not something important to see the doctor for	230 (45.3)	106 (49.5)	124 (42.2)	.10
	Too busy to go to the doctor	225 (44.3)	90 (42.1)	135 (45.9)	.42
	Has other things to do	216 (42.5)	91 (42.5)	125 (42.5)	.99
	Would try some herbs first	179 (35.2)	68 (31.8)	111 (37.8)	.19
	Financial hardship that will not allow seeing the doctor	161 (31.7)	87 (40.7)	74 (25.2)	<.001
	Difficulty arranging transports to the doctor	116 (22.8)	64 (29.9)	52 (17.7)	.001
	Family or husband would refuse their wife being examined by a male doctor	96 (18.9)	38 (17.8)	58 (19.7)	.65
	Family or husband would refuse the visit to the doctor	47 (9.3)	22 (10.3)	25 (8.5)	.54

Abbreviation: WBJ, West Bank and Jerusalem.

On the multivariable analysis, displaying a fair or good awareness of BC symptoms had no association with the total number of the reported barriers. In addition, there was no association between the awareness level and the reported number of each of emotional, service-related, or practical barriers (Table 6).

**TABLE 6:** Multivariable Logistic Regression Analyzing the Association Between Having at Least the Median Number of Barriers to Seek Medical Advice for a Possible BC Symptom and Participant Characteristics

Characteristic	All Barriers		Emotional Barriers		Service Barriers		Practical Barriers	
	AOR (95% CI)	*P*	AOR (95% CI)	*P*	AOR (95% CI)	*P*	AOR (95% CI)	*P*
Awareness of BC symptoms								
Poor	Ref	Ref	Ref	Ref	Ref	Ref	Ref	Ref
Fair	0.93 (0.56 to 1.55)	.79	1.26 (0.76 to 2.10)	.37	1.08 (0.65 to 1.79)	.77	0.68 (0.39 to 1.18)	.17
Good	1.10 (0.63 to 1.94)	.73	1.28 (0.73 to 2.25)	.40	0.94 (0.53 to 1.66)	.84	0.94 (0.50 to 1.75)	.84

NOTE. All analyses were adjusted for age group, menarche, educational level, occupation, monthly income, marital status, residency, having a chronic disease, knowing someone with cancer, and site of data collection. The outcome in all models is binary with the median number of barriers in each category used as the cutoff.

Abbreviations: AOR, adjusted odds ratio; BC, breast cancer; Ref, reference.

## DISCUSSION

The results of this study indicated that the proportion of participants who would seek medical advice immediately varied for different BC symptoms. Furthermore, the study found that exhibiting good awareness of BC symptoms was positively associated with a higher likelihood of seeking medical advice within a week for all BC symptoms. Interestingly, there was no association between increasing levels of BC awareness and reporting any specific barrier.

Similar to this study, other studies also found that women were more likely to seek help early for symptoms of a breast lump or thickening in the axilla.^29,30^ By contrast, other symptoms did not trigger immediate help-seeking behavior in the same way as symptoms associated with a mass.^29-31^ This may indicate that women associate a mass in the breast with cancer while other symptoms appear to be less strongly linked to cancer, which might be due to a gap in knowledge and awareness of BC symptoms.^13^ Therefore, educational campaigns should focus and emphasize these less familiar BC symptoms to increase awareness of all potential BC symptoms.^29,30^

Participants from the WBJ exhibited more immediate help-seeking behavior if they noticed any possible BC symptoms than those from the Gaza Strip. This difference may be attributed to various factors, including the presence of practical and service barriers that are more pronounced in Gaza, such as challenges in accessing health care, limited availability of female breast surgeons, and financial burdens associated with medical care. The health sector in the Gaza Strip is lacking adequate infrastructure and limited training opportunities for medical staff because of its geopolitical situation dominated by isolation and movement restrictions.^32^ Consequently, both residents and the health care system in the Gaza Strip encounter more constrained resources, impeding access to treatment for patients and training of medical staff compared with the WBJ.^33^ These difficulties are reflected in the results of this study.

Conversely, participants from the Gaza Strip were more likely to display good awareness of BC symptoms than those from the WBJ. This may be related to the fragile health system in the Gaza Strip. The relatively lower resources in the health sector and the presence of several practical and service barriers that women in the Gaza Strip more often encounter compared with those in the WBJ may have driven them to educate themselves more about health topics, including BC.^10,13^ In addition, there is a higher rate of unemployment among women in the Gaza Strip compared with those residing in the WBJ.^23^ This may have given women from the Gaza Strip more time and opportunities to search and read more about BC.^15^ Moreover, the WBJ has a more extensive geographical diversity compared with the Gaza Strip, with a higher proportion of women residing in rural areas, which constrains their accessibility to health care centers to interact with health care professionals and enhance their health literacy.^13,34^

The time elapsed between the onset of symptoms and the initial visit to a doctor for symptom evaluation was found to have a greater impact on women's health compared with the time between the doctor's appointment and the initiation of management.^35-37^ Low awareness of cancer symptoms is considered one of the major factors contributing to delayed presentation.^38-40^ Our findings indicate that promoting BC symptom awareness through health educational programs focusing on all the BC symptoms, including the nonspecific symptoms, might encourage women to seek medical advice earlier. Therefore, in resource-constrained settings such as Palestine, it may be more efficient and cost-effective to prioritize initiatives aimed at increasing awareness among the population, rather than primarily focusing on strengthening health care systems, which often require significant financial investments.^29^

Similar to previous studies, emotional barriers including feeling worried about what a doctor might find and feeling scared were the most reported barriers in this study.^11,12^ In low- and middle-income settings such as Palestine, the outcomes of BC treatment are usually worse when compared with high-income settings but also the symptom burden among BC sufferers and lack of support in end-of-life care might add to delayed help seeking and increase emotional barriers.^2,41,42^ This highlights the need for wider educational interventions that also target men and encourage family engagement in health decision making, including early help seeking to avoid delays to diagnosis.^43^

In this study, an important service barrier was the small number of female doctors in the Palestinian Ministry of Health facilities (20.4% female doctors and 79.6% male doctors)^44^ suggesting that women prefer female doctors to examine, investigate, and treat BC symptoms, which is consistent with previous studies.^10,12^ Increasing the number of female doctors within this field should be a priority to facilitate early help seeking of women with BC symptoms.^45^

In this study, there was no significant association between increasing awareness of BC symptoms and reporting less barriers of any type. Future educational interventions should be directed to overcome and mitigate these barriers by highlighting potential curability of BC if detected early. In addition, improvement in provision and access to BC screening might further increase early detection and reduce barriers to early presentation.^42^ A previous study from the Gaza Strip showed a 3-year survival rate of 76.1%, a 5-year survival rate of 65.1%, and a 10-year survival rate of only 51.9% after a BC diagnosis.^46^ Furthermore, a previous report from the Gaza Strip found that of 178 patients with BC, 107 (60.1%) were diagnosed with regional metastasis, of whom 53.3% died from the disease during the follow-up period.^46^ Therefore, ultimately, reducing the proportion of women who present with advanced disease stages is important to improve outcome for patients with BC in Palestine.^42^

The use of a convenience sample in this study introduces limitations regarding the generalizability of the findings to the entire population of women in Palestine. However, the large sample size and the inclusion of participants from diverse locations across Palestine help to mitigate this limitation. The exclusion of visitors to oncology departments, as well as individuals with medical backgrounds, may have resulted in a reduced number of participants with presumed good awareness of BC. However, this exclusion was intentional to ensure that the study primarily assessed the public awareness of BC rather than the knowledge of individuals already engaged with this health problem. Furthermore, it is important to note that this study focused on women who did not experience actual BC symptoms but instead examined their perceived knowledge and anticipated reactions to symptoms. It is possible that participants' actual response to experiencing symptoms may differ from their anticipated reactions.

In conclusion, the nature of BC symptoms had an impact on help-seeking behaviors. Lump symptoms were associated with shorter times to seek medical advice. Having good BC symptom awareness levels was associated with an increase in the likelihood of seeking early medical advice. Emotional barriers were the most commonly reported barriers. Participants' BC awareness level was not associated with reporting barriers to early presentation. Improving BC symptom awareness and mitigating barriers to early presentation might further facilitate timely diagnosis with BC symptoms and eventually decrease BC overall morbidity and mortality in Palestine.

## DATA SHARING STATEMENT

The dataset used and analyzed during the current study is available from the corresponding author on reasonable request.
